# Therapeutic Hypothermia for Neonatal Encephalopathy in Low- and Middle-Income Countries: A Systematic Review and Meta-Analysis

**DOI:** 10.1371/journal.pone.0058834

**Published:** 2013-03-19

**Authors:** Shreela S. Pauliah, Seetha Shankaran, Angie Wade, Ernest B. Cady, Sudhin Thayyil

**Affiliations:** 1 Academic Neonatology, Institute for Women's Health, University College London, London, United Kingdom; 2 Neonatal/Perinatal Medicine, Wayne State University School of Medicine, Children's Hospital of Michigan and Hutzel Women's Hospital, Detroit, Michigan, United States of America; 3 Paediatric Epidemiology and Biostatistics, Institute of Child Health, University College London, London, United Kingdom; 4 Medical Physics and Bioengineering, University College Hospital NHS Foundation Trust, London, United Kingdom; Hôpital Robert Debré, France

## Abstract

Although selective or whole body cooling combined with optimal intensive care improves outcomes following neonatal encephalopathy in high-income countries, the safety and efficacy of cooling in low-and middle-income countries is not known.

**Objective:**

We performed a systematic review and meta-analysis of all published randomised or quasi-randomised controlled trials of cooling therapy for neonatal encephalopathy in low-and middle-income countries.

**Results:**

Seven trials, comprising a total of 567 infants were included in the meta-analysis. Most study infants had mild (15%) or moderate encephalopathy (48%) and did not receive invasive ventilation (88%). Cooling devices included water-circulating cooling caps, frozen gel packs, ice, water bottles, and phase-changing material. No statistically significant reduction in neonatal mortality was seen with cooling (risk ratio: 0.74, 95% confidence intervals: 0.44 to 1.25). Data on other neonatal morbidities and long-term neurological outcomes were insufficient.

**Conclusion:**

Cooling therapy was not associated with a statistically significant reduction in neonatal mortality in low-and middle-income countries although the confidence intervals were wide and not incompatible with results seen in high-income countries. The apparent lack of treatment effect may be due to the heterogeneity and poor quality of the included studies, inefficiency of the low technology cooling devices, lack of optimal neonatal intensive care, sedation and ventilatory support, overuse of oxygen, or may be due to the intrinsic difference in the population, for example higher rates of perinatal infection, obstructed labor, intrauterine growth retardation and maternal malnutrition. Evaluation of the safety and efficacy of cooling in adequately powered randomised controlled trials is required before cooling is offered in routine clinical practice in low-and middle-income countries.

## Introduction

Neonatal encephalopathy occurs in 1 to 3 per 1000 live births in high-income countries, and in up to 20 per 1000 live births in low and middle-income countries. Recent meta-analysis of therapeutic cooling trials conducted in high-income countries show that whole-body, or selective, head cooling reduces mortality (risk ratio (RR) 0.78; (95% confidence intervals (CI) 0.66 to 0.93), and improves survival with normal neurological outcome after neonatal encephalopathy (RR: 1.53, (95% CI: 1.22 to 1.93)[1]. Cooling is also cost-effective in high-income countries [2], and its protective effect persists into later childhood[3]. Cooling therapy is now widely offered as standard treatment for neonatal encephalopathy in high-income countries [4].

The burden of neonatal encephalopathy in low-and middle-income countries is far higher than in high-income countries, and it accounts for approximately one million deaths annually [5]. Although the health impact of cooling therapy may be substantial, there are a number of concerns about the safety and efficacy of cooling in low-and middle-income countries. Firstly, cooling is combined with optimal tertiary neonatal intensive care in high-income countries. Most low-and middle-income country neonatal units do not have such facilities, and care may be sub-optimal. Secondly, in low-and middle-income countries the therapeutic time-window for administering beneficial cooling may be already passed due to delayed hospital admissions, prolonged or obstructed labor, lack of neonatal transport facilities, and frequent occurrence of intrauterine growth retardation. Thirdly, the incidence of perinatal sepsis is far higher in low-and middle-income countries, than in high-income countries, and neonatal sepsis may masquerade as encephalopathy. The latter is particularly worrying considering the strong separate epidemiological associations between increased mortality and sepsis and hypothermia [6]. Finally, expensive servo-controlled cooling equipment used in high-income countries is unsuitable for low-and middle-income country use [7].

We performed a systematic review, and meta-analysis of the published literature on the safety and efficacy of cooling therapy in low-and middle-income countries.

## Methods

We included all randomised, or quasi-randomised controlled trials comparing either selective head or whole body cooling (initiated within 6 hours of birth, and continued for at least 48 hours), with standard care, in term or near term infants with neonatal encephalopathy consequential to perinatal asphyxia.

Asphyxia was considered if at least one of the following criteria was met: Apgar score ≤5 at 5 min, cord or arterial blood pH ≤7.1, base deficit ≥12 mmol/L within the first hour of life, or ongoing resuscitation or mechanical ventilation at five minutes of life. Neonatal encephalopathy was defined by detailed neurological examination performed before randomisation. Studies without a standard care arm were included in the systematic review, but were excluded from the meta-analysis.

The primary outcomes were (i) neonatal mortality and (ii) moderate or severe neurodevelopmental disability at ≥18 months of age. Secondary outcomes were blood infections within the first week of life, coagulopathy or thrombocytopenia requiring blood products, respiratory failure requiring ventilator support, and hypotension requiring intervention.

### Search strategy and data analysis

We used standard Cochrane Neonatal review group (Issue 2, 2007) [8] methodology for literature search, data extraction, quality assessment and meta-analysis. We searched Medline, Embase, Cochrane Central Register of Controlled Trials (January 1995 to November 2012). We used the following search terms–‘hypoxia-ischemia’, ‘newborn’, ‘hypothermia-induced’, and ‘developing countries’. We also examined expert reviews including cross-references, abstracts, conference proceedings, and used expert informants. No language restrictions were applied.

Two review authors (SSP/ST) independently identified the studies to be included, extracted the data, and assessed the study quality based on allocation concealment, blinding of outcome assessment, adherence to intention to treat analysis, and completeness and quality of follow up [8].

The effect of cooling therapy in settings that lack basic neonatal care may be very different to those with good neonatal care. Hence, we sub-grouped the studies based on the quality of neonatal intensive care support (Level I, II and III) [9],and gross national income of the country (GNI) using World Bank economic classification (Upper middle income, Lower middle income, Low income) [10] for minimising clinical heterogeneity. We used a random effects model for meta-analysis because of the clinical heterogeneity of included studies. Statistical heterogeneity was quantified using the I^2^ test (RevMan version 5.1.4; Copenhagen).

We examined publication bias using a funnel plot [11]. A funnel plot graphically checks the existence of publication bias in systematic reviews and meta-analyses. It assumes that the largest studies will have results near the average, and results from small studies will be spread on both sides of the average. Variation from this assumption can indicate publication bias.

## Results

**Figure 1:**
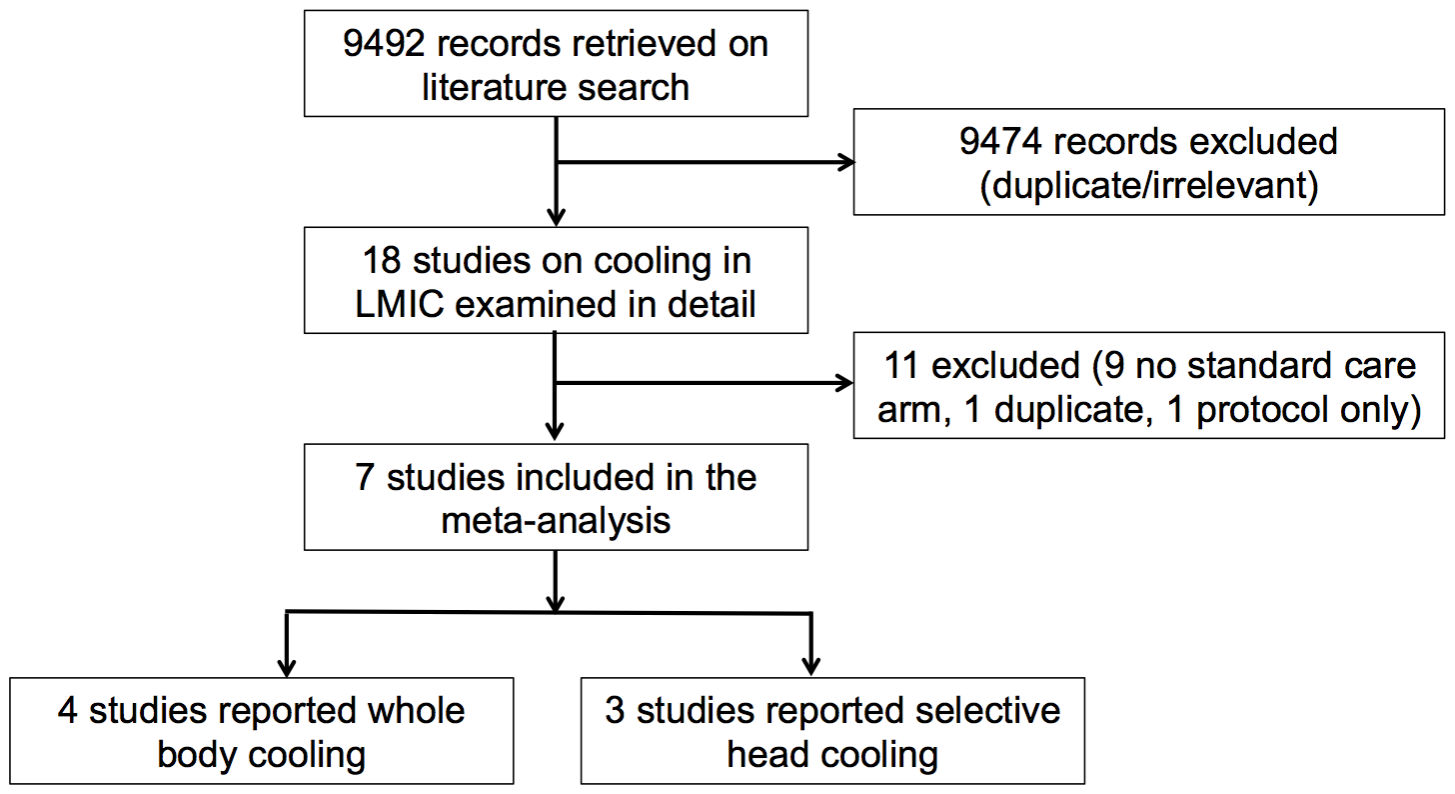
Flow chart.

We identified a total of 18 studies on cooling therapy in low-and middle-income countries, of which 11 were excluded (9 case series, 1 duplicate publication, 1 reported study protocol only). Thus we analysed the data on 567 infants recruited to seven clinical trials (3–selective head cooling, 4–whole body cooling; Figure 1). Details of excluded studies are given in Table 1.

**Table 1:** Characteristics of excluded studies.

Excluded studies	Country	Cooling method	Device	N	Comments	Reasons for exclusion
Horn[19]	South Africa	Selective head cooling	Frozen gel packs	4	Study prematurely stopped due to wide temperature fluctuations.	Case series
Horn[36]	South Africa	Selective head cooling	Frozen gel packs	5	Pilot study of selective head cooling with frozen gel packs around head	Case series
Horn[17]	South Africa	Selective head cooling	Servo controlled fan	10	Excessive shivering reported in the cooled infants.	Case series
Thomas[16]	India	Whole body cooling	Frozen gel packs	20	Recruited infants were ≥35 weeks gestation and mean rectal temperature during cooling was 32.9±0.11°C.	Case series
Rajhans[37]	India	Whole body cooling	Blanketrol II	5	Only two babies completed cooling for 72 hours	Case series
Tan[24]	Uganda	Whole body cooling	Water bottles	19	One year follow up of previously recruited infants from a cooling trial.	Duplicate data
Robertson[23]	Uganda	Whole body cooling	Water bottles	56	Study protocol of a previously published cooling trial.	Protocol only
Horn[38]	South Africa	Selective Head Cooling	Frozen gel packs	14	Active rewarming using a radiant warmer	Case series
Thomas[16]	India	Whole body cooling	Frozen gel packs	14	Long term follow up of a previous case series on whole body cooling. Adverse outcome seen in 3 (2 deaths, 1 developmental delay) of the 14 infants (out of 20) followed up till 18 to 24 months of age	Case series
See [39]	Malaysia	Whole body cooling	Ambient temperature	17	Case series that mainly recruited Stage 2 NE infants, cooled by manipulating environmental temperature; report no neurological deficit in 14/15 stage 2 NE babies.	Case series
Li [40]	China	Whole body cooling	ND	93	Hypothermic induced within 10 hours, maintaining rectal temperature 33.5°C for 72 hours. No difference in primary outcome of death or moderate-to-severe disability whether hypothermia was started at 6 hours or 6 to 10 hours.	Hypothermia induced within 10 hours of age.Study details unclear.

ND, not described; NE, neonatal encephalopathy.

The study population included only full term infants (born at ≥37 weeks gestation). The inclusion criteria for most of the studies were low Apgar score at 5 minutes and evidence of encephalopathy within 6 hours after birth (Table 2). Four studies used modified Sarnat staging and 2 used Thompson encephalopathy score [12] to define encephalopathy; details of neurological examination were not mentioned in 1 study. Two studies [13], [14] used only clinical inclusion criteria and did not include blood tests (pH or base excess) to characterise the asphyxial event. The mode of death (withdrawal of life support versus spontaneous death) was not explicitly described in many studies. Characteristics of the in-trial population are given in Table 3.

**Table 2:** Major inclusion and exclusion criteria of studies included in the meta-analysis.

Akisu[20]	Lin[21]	Zhou[22]	Robertson[13]	Thayyil[14]	Bharadwaj[15]	Bhat[18]
Inclusion criteria						
5 min Apgar <6 AND Cord pH<7.1 or base deficit >10 mmol/L AND encephalopathy	5 min Apgar <6 AND Cord pH<7.1 or base deficit >15 mmol/L AND encephalopathy	5 min Apgar <6 AND Cord pH<7 or base deficit ≤16 mmol/L AND need for resuscitation at 5 minutes of age	5 min Apgar <6 AND encephalopathy (Thompson score >5)	5 min Apgar <6 AND encephalopathy (Thompson score >5)	10 min Apgar <6 AND arterial pH≤7 or base excess ≥12 meq AND encephalopathy	10 minute Apgar <5 AND Cord pH<7 and or base deficit of >18 meq/L
Exclusion criteria						
Major congenital malformation, metabolic disorder, chromosomal abnormalities, congenital infection, transitory drug depression	Major congenital abnormalities, persistent pulmonary hypertension	Major congenital abnormalities, maternal fever >38°C, infection, rupture of membranes >18 hours or foul smelling liquor, other encephalopathy	Apnoea or cyanosis, absent cardiac output >10 min	Major congenital malformations, Imminent death at time of randomisation	Major congenital abnormalities, no spontaneous respiration by 20 min, out born babies	Not described

**Table 3:** Characteristics of the in-trial population of studies included in the meta-analysis.

	Akisu[20]		Lin[21]		Zhou[22]		Robertson[13]		Thayyil[14]		Bharadwaj[15]		Bhat[18]	
	HYPO	STD	HYPO	STD	HYPO	STD	HYPO	STD	HYPO	STD	HYPO	STD	HYPO	STD
**Number of babies**	11	10	32	30	138*	118*	21	15	17	16	62	62	20	15
**Birth weight (g)**	3410 (575)	3270 (520)	3310 (470)	3430 (520)	3360 (483)	3299 (421)	3300 (550)	3200 (268)	2977 (402)	2890 (467)	2967 (380)	2899 (363)	NA	NA
**Gestation (weeks)**	39.3 (1.4)	39.1 (0.9)	38.7 (1.3)	39.1 (1.6)	NA	NA	38 (1.5)	38 (1.4)	38 (1.2)	38.9 (0.8)	39.8 (1.4)	40 (1.4)	NA	NA
**Apgar score 5 min**	4.3 (1)	4.1 (1)	3 (1)	3 (1)	NA	NA	4.7	5.2	4.3 (0.9)	4.5 (1.0)	NA	NA	NA	NA
**Apgar score 10 min**	NA	NA	NA	NA	NA	NA	NA	NA	6 (1.5)	7.7 (1.3)	5.34 (1.4)	5.26 (1.2)	NA	NA
**Mild NE (n–%)**	0	0	7 (21)	7 (23)	21 (15.2)	18 (15.2)	5 (23.8)	4 (26.6)	9 (52.9)	9 (56.3)	NA	NA	NA	NA
**Moderate NE (n–%)**	7 (63)	5 (50)	16 (50)	15 (50)	41 (29.7)	41 (34.7)	10 (47)	10 (66.6)	6 (35.3)	5 (31.3)	55 (88.7)	54 (87)	NA	NA
**Severe NE (n–%)**	3 (27)	3 (30)	7 (21)	6 (20)	38 (27.5)	35 (29.6)	6 (28.5)	1 (6.6)	2 (11.8)	2 (12.5)	7 (11.3)	8 (13)	NA	NA
**Ventilation (n–%)**	NA	NA	4 (12.5)	5 (16)	16 (11.6)	22 (18.6)	0	0	4 (23.5)	2 (12.5)	10 (16.1)	11 (17.7)	NA	NA
**Mortality (n–%)**	0	2 (20)	2 (6.3)	2 (6.6)	31 (22.4)	46 (38.9)	7 (33)	1 (6.6)	4 (23.5)	2 (12.5)	3 (4.8)	6 (9.7)	3 (15.0)	5 (33.3)

All data are mean (SD) unless specified otherwise

HYPO, Hypothermic arm; STD, Standard care arm; NA, not available; NE, neonatal encephalopathy.

* Original number recruited into cooled and standard care arms. Nineteen infants in the cooled arm were then excluded and a further 19 lost to follow up. Two infants in the standard care arm were subsequently excluded and a further 22 lost to follow up. Thus the authors reported outcome data on 100 and 94 standard care infants.

### Cooling devices

Most studies used indigenous low technology cooling methods. For whole-body cooling these included ice and frozen gel packs (n = 2) [15], [16], cooling fan (n = 1) [17], water bottle (n = 1) [13], and phase changing material (n = 1)[14]. The cooling method was not reported in one study [18]. Selective head cooling used ice around the head (n = 1) [19] or circulating-water head caps (n = 3) [20]–[22].

Target rectal temperatures were 33 to 34°C [13]–[16], [18] for whole-body cooling and 36 to 36.5°C [19]–[22] for selective head cooling. Detailed temperature profiles and re-warming methods are given in Table 4. Control arm hyperthermia was not reported in any study; however, one study noted hyperthermia during re-warming of the cooled infants [21]. No data were available on additional resource implications or nursing input required to administer the cooling therapy.

**Table 4:** Cooling methods used in the studies included in the meta-analysis.

	Akisu[20]	Lin[21]	Zhou[22]	Robertson[13]	Thayyil[14]	Bharadwaj[15]	Bhat[18]
**Country**	Turkey	China	China	Uganda	India	India	India
**Gross National Income**	Upper middle	Upper middle	Upper middle	Low	Lower Middle	Lower Middle	Lower Middle
**Cooling method**	SHC; water cooling caps	SHC; water cooling caps	SHC; water cooling caps	WBC; water bottles	WBC: phase changing mattress	WBC: frozen gel packs	WBC: device unclear
**Target temperature (°C)**	Tympanic 33.5 to 33; Rectal 36.5 to 36	Nasopharyngeal 34 to 35	Rectal 34.5 to 35	Rectal 33 to 34	Rectal 33 to 34	Rectal 33.7	Skin and rectal 33.5
**Cooling duration (hours)**	72	72	72	72	72	72	72
Rewarming*	Active	Passive/Active**	Passive	Passive	Passive	Active	NA
**Age at cooling (min)**	114 (60)	240	246 (72)	115	264	216	NA
**Temperature at randomisation (°C)**	NA	NA (hypothermic)	NA	33.6	35.2	34.7	NA

SHC: Selective head cooling; WBC: Whole body cooling; NA, not available.

### Outcome measures

**Figure 2:**
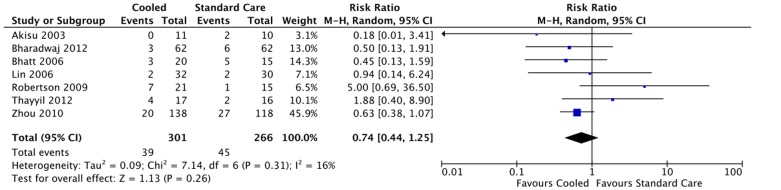
Effect of cooling on neonatal mortality.

Primary outcome (neonatal mortality) was available from a total of 567 infants recruited to the seven randomised controlled trials [13]–[15], [18], [20]–[22]. The pooled data showed no difference in neonatal mortality between the cooled and standard care infants (RR: 0.74 (95% CI: 0.44 to 1.25) (Figure 2). However, the confidence interval was wide and not incompatible with results for high-income countries. There was no evidence of significant statistical heterogeneity (I^2^ = 16%; p = 0.26). Data on encephalopathy stage was available from six trials – 15% of the infants had mild, 48% moderate and 22% severe encephalopathy.

**Figure 3:**
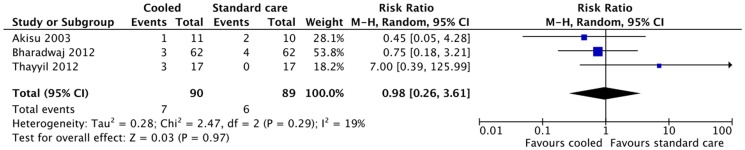
Effect of cooling on neonatal sepsis.

Three studies reported culture positive neonatal blood infections; again no significant difference was seen in infection rates (RR 0.98 (95% CI 0.26 to 3.61) (Figure 3) [15], [20], [21], although the confidence intervals were wide. The largest study excluded cases at risk of early onset neonatal sepsis [23].

Four studies reported the use of respiratory support; only 12% of infants were ventilated. Although, sedation was not routinely given to all infants undergoing cooling therapy, some studies used morphine [15], phenobarbitone [14], [21] or chloral hydrate [14] when babies were distressed.

The data were inadequate to examine other important short-term secondary outcomes like coagulopathy, respiratory failure, and hypotension. Zhou reported two cases with scalp edema and scleroderma in their study using cooling caps [22].

Three studies reported long term neurodevelopmental outcomes. Bharadwaj and Bhat reported adverse neurodevelopmental outcome at age six months in 107 (82%) of the 130 infants in their trial. Two cooled infants and 12 standard care infants had developmental delay (Baroda developmental screening test) [15]. Tan et al. examined long-term outcomes at age one year for 19 (53%) of the 36 infants in their cooling trial. Specific assessment details were not reported [24]. Zhou et al. reported 18-month outcome data on 194 (76%) of the 256 infants they recruited: severe disability in the cooled group (11/119) was similar to that in age-matched controls (19/116); RR: 0.56 (95% CI: 0.28 to 1.3) [22], although again confidence intervals were wide and differences of clinical importance could not be discounted.

Three trials were in upper mid income countries [20]–[22], three in lower middle income countries [14], [15], [18], and one in a low-income country [13]. The low-income country trial reported five times higher mortality in the cooled infants (RR: 5, 95% CI: 0.7 to 37) [13] but this difference was not statistically significant. Data were not available to perform sub group analysis based on the quality of neonatal intensive care.

One study used quasi-randomisation based on whether recruitment was on an odd or even calendar day [21]; five used randomisation with sealed envelopes, and one used computerized randomisation. All except one were small pilot (phase II) studies. The results of the meta-analysis were heavily influenced by the largest study [22], which was of poor quality. Although this was the only phase III trial and used computerised randomization; there was no assessor blinding, sealed envelopes were used on site, and there were post randomization exclusions that may have biased results. The study recruited 256 cases; 138 were allocated to selective head cooling and 118 to standard care. Nineteen infants were then excluded from the selective head cooling group and two from the standard care group, before starting intervention. A further 19 (16%) infants in the selective head cooling group, and 22 (19%) infants in the standard care group were lost to follow up (Table 5).

**Table 5:** Quality of included studies.

	Akisu[20]	Lin[21]	Zhou[22]	Robertson[13]	Thayyil[14]	Bharadwaj[15]	Bhat[18]
Adequacy of method of randomization	Yes (Computer generated)	No (Based on odd or even day)	Yes (Computer generated)	Yes (Computer generated)	Yes (Computer generated with minimization)	Yes (Computer generated)	Not known
Concealment of allocation	Not clear	None	Sealed envelopes	Sealed envelopes	Software with adequate randomisation weighing	Sealed envelopes	Not known
Blinding of intervention	None	None	None	None	None	None	Not known
Trial phase	Phase II	Phase II	Phase III	Phase II	Phase II	Phase II	Phase II
Post randomisation exclusions	No	No	Yes	No	No	No	No

**Figure 4:**
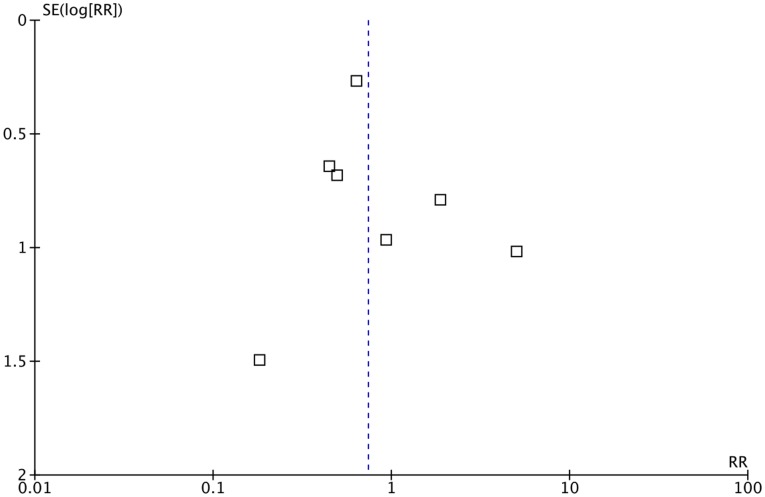
Funnel plot of the included studies.

Although no asymmetry was seen on the funnel plot (Figure 4), only 7 studies were included in the meta-analysis. Hence no definite conclusions about publication bias can be made [11].

## Discussion

The pooled data from seven low-and middle-income country randomised controlled cooling trials, including a total of 567 infants, did not show a statistically significant reduction in neonatal mortality (RR 0.74; 95% CI 0.44 to 1.25); however the confidence intervals were wide, thus clinically important benefits or harm cannot be excluded. Most studies were small and/or of poor quality, and did not evaluate long-term neurological outcomes or other important neonatal morbidities.

Our data suggests that there are a number of key differences between high-income country and low-and middle-income country cooling trials with regards to the study population, inclusion criteria, and techniques used. For example, in low-and middle-income country trials most recruited infants were reported to have mild or moderate encephalopathy. More importantly, the vast majority of the low-and middle-income country infants did not require invasive ventilatory support, suggesting that the degree of encephalopathy was much lower. This is in contrast with high-income country cooling trials, and the current practice in high-income countries where the vast majority of infants undergoing cooling also require respiratory support. Alternatively, this may be due to lack of facilities for providing optimal ventilatory support in low-and middle-income countries. Sedation was not routinely used with cooling in many studies, and this may be due to concerns about respiratory compromise.

In addition, often babies were hypothermic at randomisation, and the intrinsic hypothermia persisted for a few hours even in the control arm of some studies. This may be due to lack of radiant warmer facilities. Furthermore, unlike high-income country cooling trials, no hyperthermia was reported in the standard care infants. This may have diluted the net treatment effects of cooling.

Cooling was administered using a number of low-technology devices; this included ice [19], fans [17], frozen gel packs [15], water bottles [13], and phase changing material [14]. Selective head cooling was more problematic with marked temperature fluctuations, and hyperthermia during rewarming [21]. None of the studies reported economic aspects, or any additional nursing input required to use these cooling devices.

It is unclear whether the apparent lack of treatment effect, seen in our meta-analysis can be attributed to the use of such low technology devices, and possible inadvertent over or under cooling. Although, one high-income country cooling trial had previously reported the use of ice for whole body cooling [25], this was within the setting of a well-resourced tertiary intensive care unit with one-to-one nurse-to-infant ratio. It is possible that ice cooling without such rigorous nursing monitoring may be potentially dangerous and not neuroprotective during inadvertent temperature fluctuations [19].

The lack of treatment effect (i.e. reduction in mortality) may be also due to the heterogeneity and methodological weakness of the studies included in this meta-analysis. Such a possibility cannot be excluded due to wide confidence intervals of the pooled data. Alternatively, there may be a genuine lack of treatment effect of hypothermia in these settings. There are several arguments that could support this hypothesis. For example, medically induced hypothermia without optimal intensive care may be harmful. It is possible that mechanical ventilation was underused or not optimally monitored in these settings. High concentrations of oxygen [26] and lack of adequate sedation [27] might have also negated the neuroprotective effects of hypothermia.

Another major concern of cooling therapy in low-and middle-income country relates to the extensive literature on the association of increased mortality with hypothermia, and a potential worsening of sepsis with cooling [28]. The prevalence and profile of perinatal sepsis (gram negative infections versus group B streptococci) in LMIC is also different to that of high-income countries [29]. Although, our meta-analysis does not suggest an increase in blood stream infection with sepsis, confidence intervals were wide, and therefore potential for harm cannot be excluded. Moreover, the largest low-and middle-income country cooling study excluded infants at high risk of early onset neonatal sepsis. In addition, underlying maternal malnutrition, fetal growth restriction, and obstruction of labor may co-exist with hypoxia-ischemia in low-and middle-income countries, all of which may influence the neuroprotective effects of cooling therapy [30].

The lack of outcome data in many studies may be due to the difficulty in undertaking long term follow-up in these settings. Attrition rate in a cooling trial in sub Saharan Africa was particularly high, and only 53% of the infants could be followed up at one year of age [31].

A recent National Institute of Child Health and Human Development workshop of cooling therapy experts concluded that the safety and efficacy data on cooling from high-income countries should not be extrapolated to low-and middle-income countries [32]. Despite this, many clinicians have already offer cooling as a clinical tool in low-and middle-income countries. A recent South African survey reported that over 50% of clinicians offered cooling in clinical practice [33]. Our meta-analysis suggests that current data is insufficient to exclude significant harm or benefits of cooling therapy in low-and middle-income countries, and therefore cooling therapy should be considered experimental and should be offered only in the context of a rigorous randomised controlled trial. Clearly, such studies should be conducted only in settings where there is good basic neonatal care. Indeed, all except one study in our meta-analysis appear to have been conducted in tertiary neonatal units in middle-income countries. The only trial conducted in a low income sub-Saharan neonatal unit that lacked basic neonatal care, reported five times higher mortality in the cooled infants [13]. Efforts should be focused on prevention of encephalopathy by improving social factors and access to health care, especially antenatal care in such settings [34]. Selective head cooling is far more complex to administer than whole body cooling, and offers no increased neuroprotection benefit or reduced systemic side effects. There are appears to be little justification for using selective head cooling in low-and middle-income countries.

In summary, no reduction in neonatal mortality was seen following selective head or whole body cooling after neonatal encephalopathy in low-and middle-income countries, however, confidence intervals were wide, and significant harm cannot be excluded. Although, low technology devices effectively provided whole body cooling, neuroprotective efficacy and additional resource implications of these devices remain unclear. More importantly, the clinical characteristics of encephalopathic infants in low-and middle-income countries are different to that of high-income countries, and therefore the safety and efficacy data on cooling from high-income countries cannot be extrapolated to low-and middle-income countries. Adequately powered clinical trials are required to before cooling can be considered as a therapeutic option in low-and middle-income countries. One such multi-country trial–‘HELIX’ (Hypothermia for Encephalopathy in Low Income countries) is currently being set up [35]. However, until more evidence from such trials are available, best practice guidelines for use of therapeutic hypothermia in low-and middle-income countries, jointly prepared by neonatologists in these settings and cooling experts from high-income countries may be required.
